# LPIN2 is the phosphatase dominating the penultimate step of neutral lipid biosynthesis in *Dictyostelium*

**DOI:** 10.17912/micropub.biology.001296

**Published:** 2024-10-23

**Authors:** Frederik Kappelt, Markus Maniak

**Affiliations:** 1 University of Kassel, Kassel, Hesse, Germany

## Abstract

*Dictyostelium* amoebae store surplus fatty acids from the diet in form of lipid droplets. Some of the enzymes governing neutral lipid synthesis are already known. For the phosphatidic acid-specific phosphatases, six genes were found, one of which was automatically annotated as LPIN2. Two GFP-tagged variants of LPIN2 homogeneously distribute in the cytoplasm and no organelle association was observed. *LPIN2 ^–^* mutants contain less than 17% residual amount of the major neutral lipid species, but phospholipid amounts are not obviously affected. A growth retardation on bacteria as food source may suggest that lipid droplets serve to detoxify excess free fatty acids.

**Figure 1.**
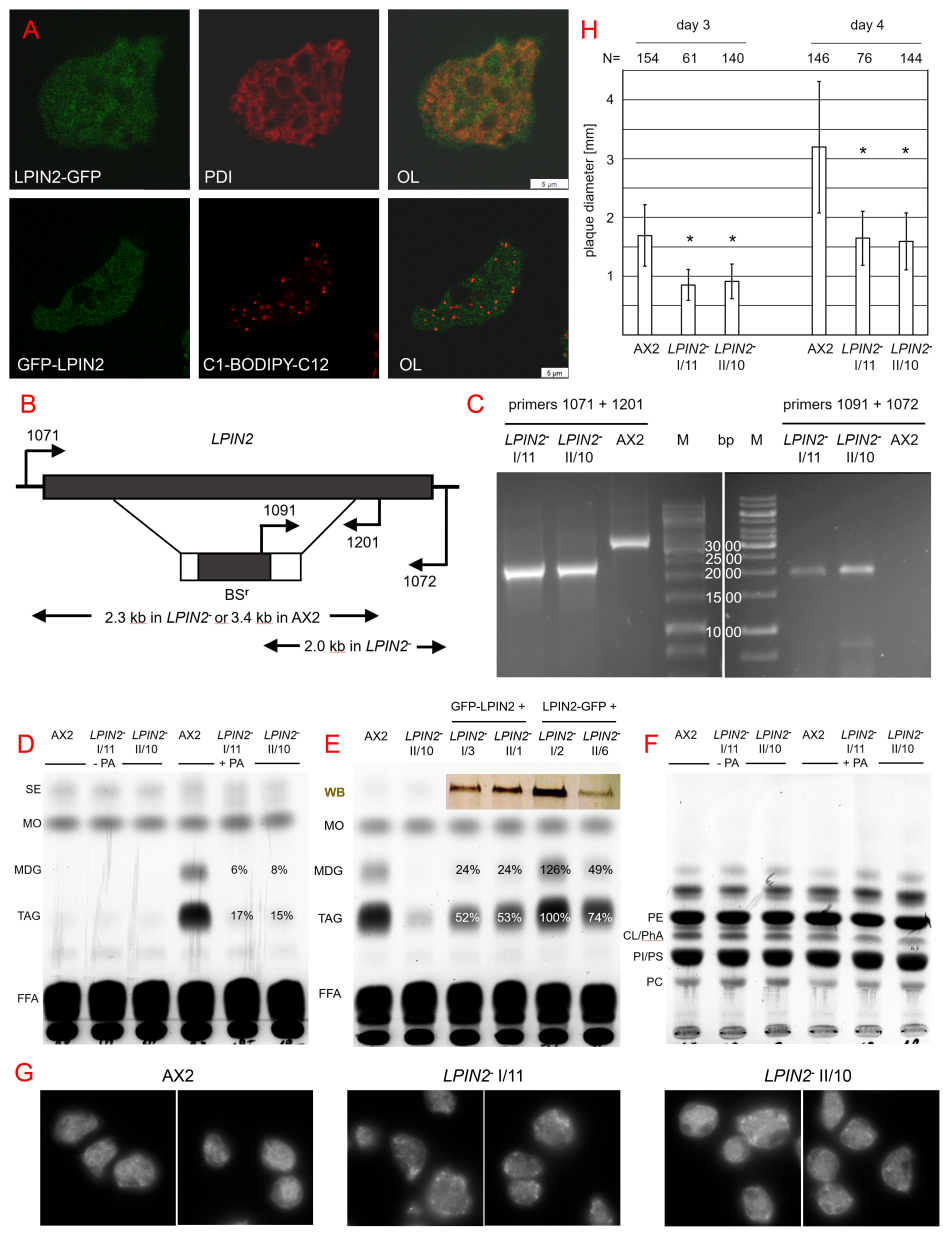
Figure 1. LPIN2 is a soluble enzyme required for neutral lipid synthesis and growth on bacteria (A) LPIN2 is a cytosolic enzyme. Cells expressing LPIN2-GPF (green) were fixed and stained with an antibody directed against the ER-resident protein-disulfide-isomerase (PDI, red). Alternatively, living cells producing GFP-LPIN2 (green), were fed for 3 hrs with a mix of palmitic acid and the fluorescent tracer C _1_ -BODIPY-C _12_ that accumulates in lipid droplets (red). One confocal section each was taken and both channels are displayed separately or as an overlay (OL). The size bar corresponds to 5 µm. (B) Diagram of the genomic *LPIN2* locus after insertion of a blasticidin S resistance cassette by homologous recombination in the coding region. Diagnostic primers are numbered and their binding sites are indicated by arrows. They reside in the coding region (grey box), the resistance gene (BS ^r^ ), or the noncoding regions (thin lines) upstream and downstream of the sequences used for targeting. The positions and sizes (kb) of expected products are indicated beneath. (C) PCR products from genomic DNA isolated from the wildtype strain (AX2) and two independently derived *LPIN2 ^-^* mutants (I/11 and II/10). Combining one primer binding 5’ upstream of the *LPIN2* coding region (1071) and one primer complementary to the LPIN2 coding region downstream of the resistance cassette (1201), a 2.3 kb fragment is amplified in the knockout mutants, whereas the size of the diagnostic fragment is 3.4 kb in the wildtype. Using a primer specific for the resistance cassette (1091) together with one that binds in the 3´ noncoding region (1072), the disrupted copy of the *LPIN2* gene can only be amplified in the mutants but not in wildtype. The relevant sizes of the DNA marker (M) are given in base pairs (bp). (D) Axenically grown cells were used as controls (-PA) or stimulated by palmitic acid addition (+PA) for 3 hrs. Thin layer chromatography (TLC) resolving steryl-esters (SE), monoalkyl-diacyl-glycerol (MDG), triacylglycerol (TAG), free fatty acids (FFA) from lipid extracts of AX2 and *LPIN2 ^-^* mutants I/11 and II/10. Methyloleate (MO) was used as a loading control. Per cent values indicate mean residual amount of MDG and TAG in mutants as compared to AX2, as determined by ImageJ-densitometry of 7 TLC plates. (E) *LPIN2 ^-^* strain II/10 was rescued by expression of GFP-LPIN2 (clones I/3 and II/1) or LPIN2-GFP (clones I/2 and II/6). Their neutral lipids were analysed under the same conditions as used in panel D (+PA), except that densitometry was based on three independent lipid extractions and TLC separations. The inset shows a western blot (WB) of GFP-tagged LPIN2 expression levels as detected by an antibody directed against GFP (F) Thin layer chromatography to analyse the relative amounts of phospholipids in the wildtype (AX2) and in *LPIN2 ^-^* mutants. The main lipids species were identified by comigration with different mixed sets of commercially available standard lipids, namely phosphatidylethanolamine (PE) cardiolipin (CL), phosphatidic acid (PhA), phosphatidylinositol (PI), phosphatidylserine, (PS), and phosphatidylcholine (PC). Otherwise cultivation conditions were as described for panel D. One typical TLC plate of at least three repetitions is shown. (G) Cells of the wildtype (AX2) or two *LPIN2* knockout strains (I/11 and II/10) were fed with palmitic acid for 3 hrs, fixed and stained with an antibody directed against PDI. Two groups of cells each were photographed on a wide-field fluorescence microscope. Fields of view are 40 µm wide. (H) Cells of AX2 and the two *LPIN2 ^-^* mutants were diluted in a thick suspension of bacteria and spread on agar plates. The diameter of the resulting plaques (in mm) on the lawn was measured on two subsequent days. Columns show mean values, and error bars represent the standard deviations derived from a total number (N=) of plaques as indicated above each column and * signifies a p value < 0.05.

## Description

*Dictyostelium discoideum* is a unicellular soil amoeba that has become a model system for studying various questions in cellular and developmental biology. Laboratory strains, such as AX2 used here, grow efficiently in liquid broth or even faster on a bacterial lawn, reflecting the nutrient density of the food internalized by macropinocytosis or phagocytosis, respectively. Surplus metabolites from bacterial food are used to produce neutral lipids, mainly triacylglycerol (TAG) (Matsuoka et al., 2003) , while this is only observed if fatty acids are added to the axenic liquid medium, resulting in the formation of fat storage organelles, so called lipid droplets (LDs) (von Löhneysen et al., 2003; Du et al., 2013) .

In the vegetative phase, *Dictyostelium* LDs mainly contain TAG, to a lesser extent the ether lipid monoalkyl-diacyl-glycerol (MDG), and traces of steryl-esters (SE) (Du et al., 2013) . Basically, all of these neutral lipids originate from the sequential activity of acyl-transferases (AT). The endoplasmic reticulum (ER)-resident enzyme GPAT transfers a first acyl-chain to glycerol-3-phosphate (GP) and cells devoid of GPAT are strongly impaired in TAG production (Kappelt et al., 2020) , whereas the enzyme FARAT initiates the pathway of MDG synthesis in the peroxisome, linking a fatty acid to the dihydroxy-acetone-phosphate backbone (Kappelt, et al., 2020) . The enzymes adding the second fatty acid to these products in *Dictyostelium* are as yet undescribed, but likely to be found among the multi-membered AGPAT gene family. For both pathways, the third fatty acid is ligated to the substrates by DGAT1, an ER-enzyme, yielding TAG and MDG, which are then packaged into LDs (Du et al., 2014) . DGAT2, an LD-resident enzyme, can replace DGAT1 in the production of TAG, but not MDG (Du et al., 2014) . However, before the DGAT-enzymes can finalize the synthesis of neutral lipids, the phosphate has to be cleaved off (yielding diacylglycerol, DAG) by a lipid-phosphatase, which we identify here.

The *Dictyostelium* genome bears six phosphatidic acid-specific phosphatases, among them one automatically annotated as LPIN2 (DDB_G0271730), because its sequence is most homologous to lipin 2 of humans and mice, which differs from lipin 1 and 3 by its tissue distribution, being also prominently expressed in the brain (Dwyer et al., 2012) . The *Dictyostelium* enzyme is 1325 amino acids long and thus about 400 residues larger than the human protein. The additional sequences are characterized by extensive homopolymer stretches of serine, threonine, glutamine and asparagine with up to 30 repetitions. Both GFP-tagged variants homogeneously distribute in the cytosol of *Dictyostelium* cells and no association is seen with the ER or LDs ( Fig. 1A ) like for other enzymes involved in neutral lipid synthesis. While human lipin 1 is associated with mitochondria and imported into the nucleus (Ren et al., 2010; Eaton, et al., 2013) where it induces the formation of nuclear LDs (Soltysik et al., 2021), the localization of lipin 2 depends on its phosphorylation status: the phosphorylated form is in the cytosol, while the dephosphorylated variant associates with membranes, where it presumably finds its substrate (Choi et al., 2012; Han et al., 2012; Barbosa et al., 2015) .

In order to generate a mutant lacking the LPIN2 protein, we replaced a 2.8 kbp long central fragment of the gene by a cassette conferring resistance to blasticidin S ( Fig. 1B ). After electroporating a linear DNA-fragment into *Dictyostelium* AX2 wildtype cells, we screened individual clones from bacterial lawns for the occurrence of homologous recombination by PCR analysis ( Fig. 1C ). Most notably, it was only among the growth-retarded small clones, where two lines with independent integration events (I/11 and II/10) were identified and subjected to further investigation.

Extracts of neutral lipids were analysed by thin layer chromatography (TLC) ( Fig. 1D ) and densitometry revealed that *LPIN2 ^-^* mutants produced about 85% less TAG and over 90% less MDG as compared to wildtype cells ( Fig. 1 c), indicating that the double-acyl-containing substrate and the mixed substrate bearing one alkyl and one acyl chain are both dephosphorylated by LPIN2. Here it is interesting to note, that deletion of the yeast lipin PAH1 also causes a reduction of 90% in the neutral lipid level, but additional deletion of three other phosphatidic acid phosphatases still does not eliminate the residual amounts of TAG (Chae et al., 2012) . In mice, lack of lipin 2 leads to anemia and neuronal defects at older age (Dwyer et al., 2012) , but a combined knockout of lipin 1 and 3 results in complete lipodystrophy (Csaki et al., 2014) as does the elimination of DGAT1 and 2 (Harris et al., 2011) .

To confirm that the neutral-lipid phenotype originates from the disruption of the *LPIN2* gene, we transformed knockout strain II/10 with either GFP-LPIN2 or LPIN2-GFP expressing plasmids and documented the reappearance of TAG and MDG again by TLC ( Fig. 1E ). Cells showing the fully rescued phenotype (I/2) expressed more LPIN2-GFP, than a strain with a partial rescue producing less LPIN2-GFP (II/6) or strains producing GFP-LPIN2 (I/3 and II/1) by western blotting (inset in Fig.1E), indicating that the rescue-effect is largely dose-dependent, although blockage of LPIN’s N-terminus seems to compromise its activity slightly.

For *LPIN2 ^-^* mutants, the first assumption would be, that the ether-form and the ester-form of the LPIN2-substrates should accumulate in the cell. However, a TLC analysis of phospholipids revealed that at the position that is occupied by phosphatidic acid (PhA) (and cardiolipin, that behaves indistinguishably under these conditions) is characterized by a mildly reduced, rather than enhanced, density of the signal ( Fig. 1F ). Interestingly, in most organisms phosphatidyl-ethanolamine (PE) and phosphatidyl-choline (PC) synthesis is based on DAG, the product of lipin. However, the amount of PC appears unaltered in the *Dictyostelium* *LPIN2 ^-^* mutants ( Fig. 1F ). PE comigrates with phosphatidyl-glycerol (PG) under the conditions used here, the former constitutes 31% of total phospholipids and PG is extremely rare 1% in *Dictyostelium* (Weeks and Herring, 1980) . Because PE-levels are also not diminished in *LPIN2 ^-^* cells ( Fig. 1F ), our observations support the notion that *Dictyostelium* either tightly controls the relative amounts of both phospholipids, or even uses an alternative pathway to synthesise them. Other phospholipids are also not conspiciously elevated, so it remains obscure where the added fatty acids remain. Because *DGAT1/DGAT2* double knockout mutants, that are also strongly impaired in the synthesis of TAG and MDG (Du et al., 2014) , form accumulations of excess ER membranes in so-called “karmellae” (Barisch and Soldati, 2017) , we stained our mutants with an antibody directed against PDI and indeed observed punctate structures in the cellular periphery, which occur less frequently in the wildtype ( Fig. 1G ).

Finally, we revisited the observation that *LPIN2* knockout mutants originated from small colonies during the clonal selection procedure. When mutant cells were plated on bacterial lawns, the plaque diameter was found to be only 50% of the AX2 wildtype value ( Fig. 1H ). Although this phenotype is strong and significant (p < 0.05), it is not as severe as the defect observed in the *DGAT1/DGAT2* double knockout mutant (Du et al., 2014) . Together these observations suggest that the ability to produce and to store neutral lipids is essential for *Dictyostelium* growing on bacteria as a substrate, most likely detoxifying excess amounts of free fatty acids in the harmless form of neutral lipids. Another feature, that both mutant strains have in common, is that after completing development in wild type-like timing, the harvested spores need many days (rather than a few hours) to germinate in bacteria-free culture medium. The reason explaining the latter observation, however, will require further investigation.

## Methods

**Cell growth.** Cells of the *Dictyostelium* AX2 strain (referred to as wildtype) and mutants constructed in this genetic background were grown at 21 ^O^ C in HL5+ medium (Formedium, UK) in shaking suspension and lipid droplet formation was induced by adding palmitic acid to a final concentration of 200 µM as described before (Du et al., 2013) .

**Molecular biology.** DNA and protein sequences for *Dictyostelium* *LPIN2* (DDB_G0271730) encoding lipin were obtained from the fully sequenced genome (Eichinger, et al., 2005) via http://dictybase.org. It was amplified from genomic DNA using primers 1069 and 1070 and ligated into pGem T-easy (Promega) to yield plasmid 1267. This plasmid was taken as a template for a second amplification with primers 1115 and 1116 to provide the gene with SalI and XhoI restriction sites. Again, the amplicon was ligated into pGemT-easy yielding plasmid 1270. Subsequently the gene was inserted via SalI and XhoI into pDNeo2a-GFP (Dubin and Nellen, 2010) resulting in a GFP-LPIN2 expressing construct (plasmid 1278). Because one base was found to be missing in the region of primer 1115, plasmid 1278 was opened with SalI, filled in with dNTPs using the Klenow enzyme, so that four base pairs were added to reconstitute the reading frame of the fusion protein, yielding plasmid 1327. To tag the LPIN2 protein on its C-terminal end, primers 1113 and 1114 were used not only to add PstI and BamHI restriction sites to *LPIN2* , but also to delete the stop codon. The amplicon was ligated into pGemT-easy for sequencing and subsequently cloned into plasmid pDNeo2a-GFP (Dubin and Nellen, 2010) , resulting in plasmid 1279.

For the insertion of a BS ^r^ -cassette into the *LPIN2* gene, plasmid 1267 was digested with HindIII and BglII deleting a 2.8 kb region of the coding section. Then a HindIII and BamHI cut BS ^r^ cassette from pLPBLP (Faix et al., 2004) was ligated in between the two remaining flanking regions of *LPIN2* . The resulting plasmid 1268 contains the 1.5 kb BS ^r^ -cassette in the same orientation as *LPIN2* . To facilitate homologous recombination, the construct was digested with EcoRI before the fragment was electroporated into *Dictyostelium* cells. Individual clones were derived by spreading cell dilutions on bacterial lawns. Plaque diameters of mutant cells were measured in a similar fashion. Primers 1071 and 1072 from the 5´ and 3´ untranslated regions, which were not part of the construct, were combined with primer 1201 in the downstream coding region, or primer 1091 binding in the BS ^r^ -cassette, respectively, to analyse the integration event in the genome. After four attempts without success, clones were searched specifically among slow growing colonies and several independent knockouts were identified, but analysis was continued with mutants I/11 and II/10.

**Lipid analysis.** The classical method of lipid preparation by Bligh and Dyer (1959) was adapted as described previously (Du et al., 2013) . Subsequently, neutral lipids were separated by thin-layer chromatography on silica plates as described by Kappelt et al. (2020). Phospholipids were analysed by the method of Vitiello and Zanetta (1978).

**Immunofluorescence experiments and GFP microscopy** were performed as described before (Maniak et al., 1995) . The distribution of the endoplasmic reticulum was shown by indirect immunofluorescence using undiluted mouse monoclonal antibody (MAb) supernatant raised against the protein-disulfide isomerase (PDI; MAb 221-64-1; Monnat et al., 1997) and detected using CY3-coupled goat-anti-mouse polyclonal secondary antibodies (Dianova). LDs were metabolically labelled with the fluorescent fatty acid analogue C _1_ -BODIPY-C _12_ (5 µM final concentration, ThermoFisher) supplemented together with 200 µM palmitic acid for 3 hr in growth medium. Images were taken as single confocal planes using a Leica TCS-SP laser scanning microscope.

## Reagents

**Primer sequences**

1069 GATAGATAGATAGAGACATATAGAAGAATGAATTATGTTG

1070 CTAAAGAGGATCTAATTTATGAAGTGGTATAACAG

1071 AAAAAATCATTGCAGGTTTGACTGTATAG

1072 GAAAAAAACAAACCACAACTTCAACAAC

1091 GAGTTGATTTCAGACTATGCACC

1113 CTGCAGAAAATGAATTATGTTGAAAAGTTATTTGACG

1114 GGATCCAAGAGGATCTAATTTATGAAGTGG

1115 GTCGACAAAATGAATTATGTTGAAAAGTTATTTGACG

1116 CTCGAGCTAAAGAGGATCTAATTTATGAAGTGG

1201 GATGCTGATACCTCTTTGGTACC
